# Bridging the gap: evaluating high TB burden country data needs to support the potential introduction of TB vaccines for adolescents and adults: a workshop report

**DOI:** 10.3389/ftubr.2024.1384036

**Published:** 2024-04-29

**Authors:** Rebecca A. Clark, Carly Young, Shaun Palmer, Erick Auma, Shelly Malhotra, Rupali Limaye, Birgitte Giersing, Lewis Schrager, Gerald Voss, Edine Tiemersma, Mike Frick, Ijeoma Edoka, Alemnew F. Dagnew, Thokozile Nkhoma, TB Vaccine Country Data Needs Group, Pei-Chun Chan, Puck T. Pelzer, Richard G. White

**Affiliations:** ^1^TB Modelling Group and TB Centre, London School of Hygiene and Tropical Medicine, London, United Kingdom; ^2^Centre for the Mathematical Modelling of Infectious Diseases, London School of Hygiene and Tropical Medicine, London, United Kingdom; ^3^Department of Infectious Disease Epidemiology, London School of Hygiene and Tropical Medicine, London, United Kingdom; ^4^Vaccine Centre, London School of Hygiene and Tropical Medicine, London, United Kingdom; ^5^South African Tuberculosis Vaccine Initiative, Institute of Infectious Disease and Molecular Medicine, Division of Immunology, Department of Pathology, University of Cape Town, Cape Town, South Africa; ^6^Stichting International AIDS Vaccine Initiative, Amsterdam, Netherlands; ^7^Stop TB Partnership Working Group on New TB Vaccines, New York, NY, United States; ^8^Division of Molecular Biology and Human Genetics, Stellenbosch University, Stellenbosch, South Africa; ^9^International AIDS Vaccine Initiative, New York, NY, United States; ^10^International Vaccine Access Center, Johns Hopkins Bloomberg School of Public Health, Baltimore, MD, United States; ^11^Initiative for Vaccine Research, World Health Organization, Geneva, Switzerland; ^12^TuBerculosis Vaccine Initiative (TBVI), Lelystad, Netherlands; ^13^KNCV Tuberculosis Foundation, Amsterdam, Netherlands; ^14^Treatment Action Group, New York, NY, United States; ^15^Health Economics and Epidemiology Research Office (HE2RO), Department of Internal Medicine, Faculty of Health Sciences, University of the Witwatersrand, Johannesburg, South Africa; ^16^School of Public Health, Faculty of Health Sciences, University of the Witwatersrand, Johannesburg, South Africa; ^17^Bill & Melinda Gates Medical Research Institute, Cambridge, MA, United States; ^18^Facilitators of Community Transformation (FACT) Malawi, Lilongwe, Malawi

**Keywords:** tuberculosis, vaccines, adolescents and adults, epidemiology, impact modeling, feasibility, acceptability

## Abstract

High tuberculosis (TB) burden countries (HBCs) need to prepare for TB vaccine implementation alongside licensure, to ensure rapid rollout. WHO policy/implementation frameworks have been created to support this effort. Using WHO frameworks, we convened a workshop to ask HBC experts about what epidemiological, impact, feasibility and acceptability data they anticipated they would need to guide TB vaccine introduction. For required data, we asked HBC and global experts which data were already available, data collection planned, or gaps. HBC experts expressed high demand for epidemiological, impact, feasibility and acceptability data, reported variable availability of existing epidemiological data, and low availability for impact, feasibility, and acceptability data. Global experts reported additional knowledge of existing data on impact, upcoming collection of infection prevalence, acceptability and feasibility data, and potential epidemiological data collection on adolescents, adults, people living with HIV, and underweight individuals. HBC and global experts made key recommendations for: a coordinated data collation, collection, analysis and sharing system; updating existing HBC health and economic impact estimates and extending impact analyses to other HBCs; demand/market forecasting; resource gap mapping; aligning delivery strategies; addressing manufacturing, procurement, delivery, and regulatory barriers; sharing potential vaccine licensure timing; incorporating TB vaccine introduction strategies into NSPs, immunization programs, and health services; collecting vaccine hesitancy, mistrust, and misinformation data; collecting adolescent/adult vaccine demand generation data, and identifying funding. Experts recommended expanding this analysis to other areas of the WHO frameworks, including more HBC stakeholders, and repeating this analysis after country and community advocacy and socialization around different vaccine candidates.

## 1 Introduction

The only globally available tuberculosis (TB) vaccine is the century-old Bacille Calmette-Guérin (BCG) vaccine. While this vaccine has efficacy in protecting against severe forms of TB disease, such as TB meningitis, in infants and young children, it is largely ineffective in adolescents and adults, among whom most disease occurs (1). There are multiple TB vaccine candidates entering or already in Phase 2b and 3 clinical trials designed to test the safety and efficacy of the vaccine candidates for use in adolescents and adults (2). To ensure new TB vaccines are available, accessible, and accepted (3), countries need to prepare for vaccine introduction in parallel to licensure. This will be critical to ensure rapid rollout.

A range of complementary efforts are underway to facilitate and prepare for the country-level introduction of new TB vaccines among adolescents and adults. The World Health Organization (WHO) has led several of these efforts, including the Evidence Considerations for TB Vaccine Policy (ECVP) guidance, which aims to provide early information on the data and evidence that are likely required to support WHO policy recommendations for the introduction of new TB vaccines intended for adolescents and adults (4). Additionally, the WHO Global Framework to Prepare for Country Introduction of New TB Vaccines for Adolescents and Adults is under development (3).

Adolescents and adults are not routinely prioritized by immunization programs in high TB burden countries, due, in part, to infrastructural and programmatic barriers to introducing vaccines among this population (5). Improved evidence and innovative strategies are likely required to identify and address the specific challenges of prioritizing new TB vaccines for adolescents and adults. Lessons from the introduction of other vaccines for adolescents and adults, such as for COVID-19 and HPV, can also inform the strategies for new TB vaccines. It is important to consider the individual country data needs and activities required to introduce and sustain the delivery and uptake of new TB vaccines. There is a need for coordination and synergy between stakeholders at the sub-national, national, regional, and global levels to inform the introduction of new TB vaccines.

We conducted a survey and convened a workshop to ask experts from countries with a high burden of TB, TB/HIV, or multidrug-resistant/rifampicin-resistant TB (MDR/RR-TB), as defined by WHO (6), what data they would need to decide whether to introduce new TB vaccines and to guide strategies for their efficient introduction. We restricted the scope to epidemiological, impact, feasibility and acceptability data. We asked high-burden country (HBC) and global experts which of these data were available and/or collection was being planned, and how gaps in currently available data could be filled. To our knowledge, this was the first time this kind of survey has been carried out—a crucial first step to inform HBC data and coordination needs.

## 2 Methods

We carried out a rapid pre-workshop online survey of a convenience sample of only HBC experts on data needs and availability, and a virtual workshop with HBC and global experts, where global experts were given a survey requesting additional knowledge on country-level data availability only. In the pre-workshop online survey, which ran between 25–28 September 2023, HBC experts were asked to identify their anticipated country-level data needs, and whether these data were currently available. The workshop, conducted on 6 October 2023, consisted of background presentations, a breakout group to discuss country and global experts' knowledge of data availability, and a breakout group to discuss country and global experts' views on the identified data gaps, and how these gaps could be filled.

**Figure 1:**
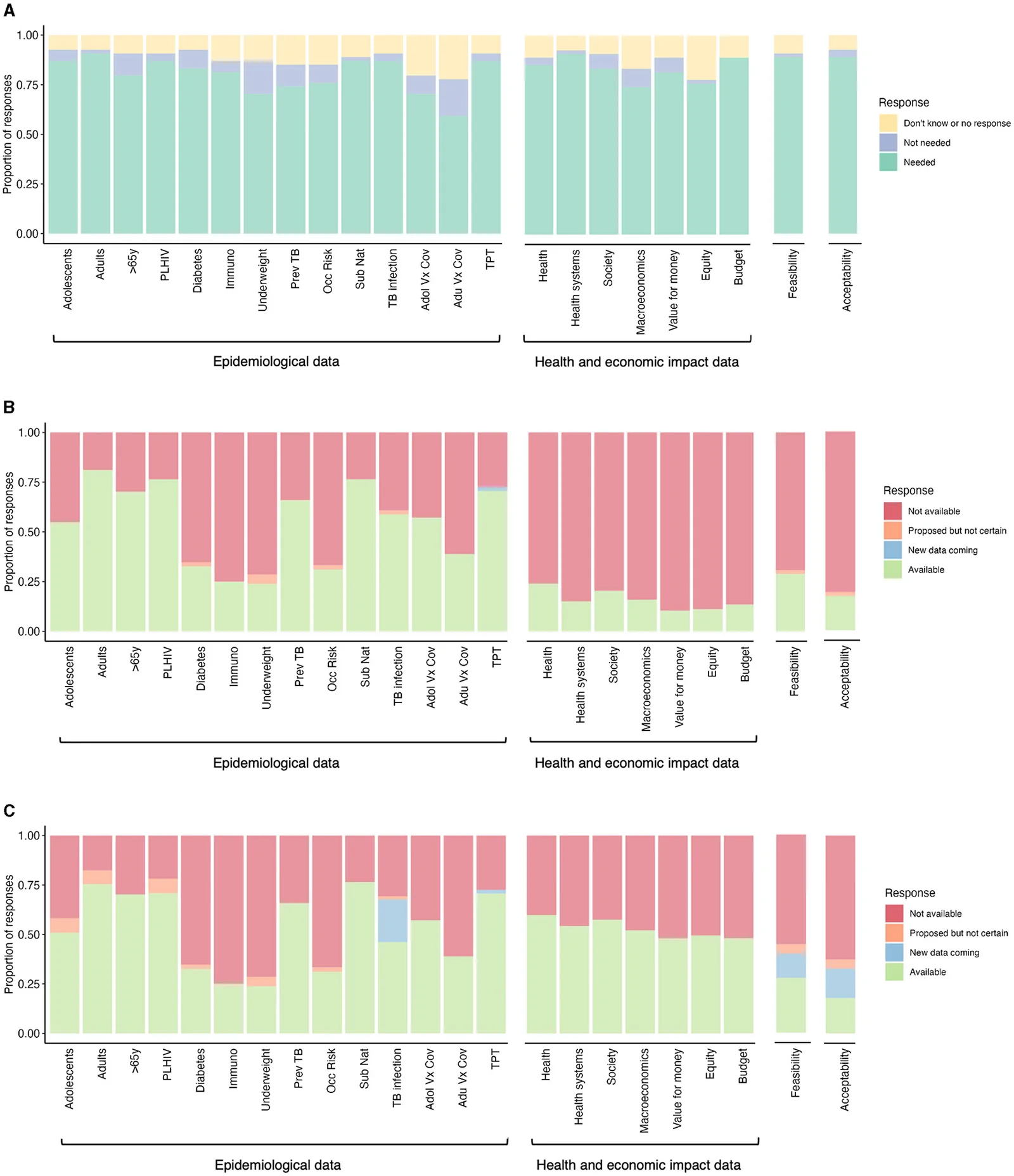
Data needs and availability to inform decision-making for new TB vaccine implementation. **(A)** Data needs expressed by High Burden Country (HBC) experts to inform decision-making for new TB vaccine implementation (*n* = 54). The perceived data availability was expressed by **(B)** HBC and global experts, and **(C)** HBC experts, global experts, and workshop leads to inform decision-making for new TB vaccine implementation [*n* varies within a range of *n* = 36–53 for **(B)** and **(C)** per category, depending on whether a respondent indicated a need in **(A)]**. PLHIV, people living with HIV; Immuno, immunocompromized individuals; Prev TB, individuals with previous tuberculosis disease; Occ risk, occupational risk; Sub nat, subnational level data; Adol Vx Cov, adolescent vaccine coverage; Adu Vx Cov, adult vaccine coverage; TPT, TB preventive therapy coverage.

The scope was restricted to data on epidemiology (potential vaccine priority groups size and TB burden, TB infection prevalence, and other vaccines and TB preventive therapy (TPT) coverage), impact (on health, health systems, society, macroeconomics, equity and budgets, and value for money of new vaccines), feasibility (practicality of vaccine implementation), and acceptability (of the vaccine to potential target populations and other key stakeholders) (Figure 1). The survey questions were restricted to these areas as these were the areas of expertise of the workshop leads. A copy of the survey provided to HBC experts is included in the Supplementary Data Sheet 1.

### 2.1 Pre-workshop survey

Potential workshop participants from high burden countries, as well as individuals with global expertise on at least one of the areas, were identified through the Working Group for New TB Vaccines (WGNV) or co-authors' networks. These invitees were encouraged to forward the invitation to any relevant individuals. Invitations to participate in the workshop were sent to 184 individuals with 101 ultimately attending. The HBC experts included representatives from National Tuberculosis Programs (NTP), National Immunization Technical Advisory Groups (NITAG), non-governmental organizations (NGOs), and National Immunization Programs (NIP). An initial email invitation was sent to HBC experts, followed by a subsequent reminder to non-respondents. Additionally, a snowballing approach was employed, allowing for the expansion of participation through recommendations and suggestions from the initial respondents. In total, 102 HBC experts from 26 countries were invited to fill in the survey.

Our survey questions were categorized into the “3As” Framework of Available, Acceptable, and Accessible (3). Questions were matched to data areas specified in the WHO ECVP (4) and aligned with data areas investigated in a recent survey conducted by SMART4TB.

### 2.2 Workshop

The opening session of the workshop featured seven short presentations. Thokozile Nkhoma, a TB survivor from Malawi, underscored the critical need for new TB vaccines in a poignant personal account, emphasizing the urgency of addressing gaps in TB prevention. Subsequently, Ijeoma Edoka (University of the Witwatersrand, South Africa), provided a summary of key epidemiological, impact, feasibility, and acceptability data needs reported by the survey of HBC experts (Figure 1). Following this perspective, Birgitte Giersing (Department of Immunization, Vaccines & Biologicals, WHO) delivered a summary outlining crucial insights from the WHO ECVP and Country Framework. Richard White (London School of Hygiene & Tropical Medicine, UK), then presented a four-country led epidemiological data collection and health and economic modeling funding proposal. Rupali Limaye (Johns Hopkins University, US), followed with a comprehensive presentation on the SMART4TB desk review, shedding light on both their planned and proposed initiatives. The session continued with Birgitte Giersing offering an update on the Boston Consulting Group/ WHO Landscape assessment. Alemnew Dagnew [Gates Medical Research Institute (GMRI), US], concluded the session with a presentation on their epidemiological data collection study.

Participants were then assigned to breakout groups based on whether they primarily were able to provide HBC-level input or global-level expertise. Those in the HBC-level breakout groups included representatives of national-level technical agencies, researchers, and program coordinators, such as NTPs. The global-level breakout groups included representatives from international global health agencies and philanthropic foundations and included representatives from the Amsterdam Institute for Global Health and Development, the Bill & Melinda Gates Foundation, the European and Developing Countries Clinical Trials Partnership, the Global TB Community Advisory Board, Gates Medical Research Institute, IAVI, KNCV TB PLUS, London School of Hygiene and Tropical Medicine, Stop TB Partnership, Treatment Action Group, the United States Agency for International Development, the University of Cape Town, Wellcome, and the World Health Organization.

The breakout sessions were moderated by experts working on TB vaccine research. The moderators were trained prior to the workshop and guided through the discussion points. Breakout groups followed a semi-structured format and were moderated by a member of the organizing group who received training prior to the workshop. The first breakout group explored participants' insights about the survey findings on data needs and availability reported on during the workshop presentations. During the second breakout group, discussions were guided by a table of prompts to facilitate active participant engagement and spontaneity. Participants would first identify a key gap and then propose activities, resources, and partners needed to address the gap, with an additional column for other insights.

## 3 Results

### 3.1 How can we meet country data needs?

The surveys on country-level data were completed by 54 respondents from 20 countries, including representatives from NTPs, NITAG, NGOs, and National Immunization Programs (NIP). The countries were: Afghanistan (one respondent), Bangladesh (2), Brazil (1), Ethiopia (2), India (8), Indonesia (3), Kazakhstan (1), Kenya (3), Kyrgyz Republic (7), Malawi (3), Mozambique (1), Philippines (3), South Africa (6), Taiwan (1), Tajikistan (1), Tanzania (3), Uganda (2), Ukraine (1), Vietnam (3), and Zimbabwe (2). The workshop was attended by 71 HBC-level participants representing 18 countries that had previously responded to the survey, and 32 global-level participants representing 19 global organizations. The two moderated breakout group sessions consisted of eight to nine groups per session, with between three and seven participants in each group.

**Figure 2:**
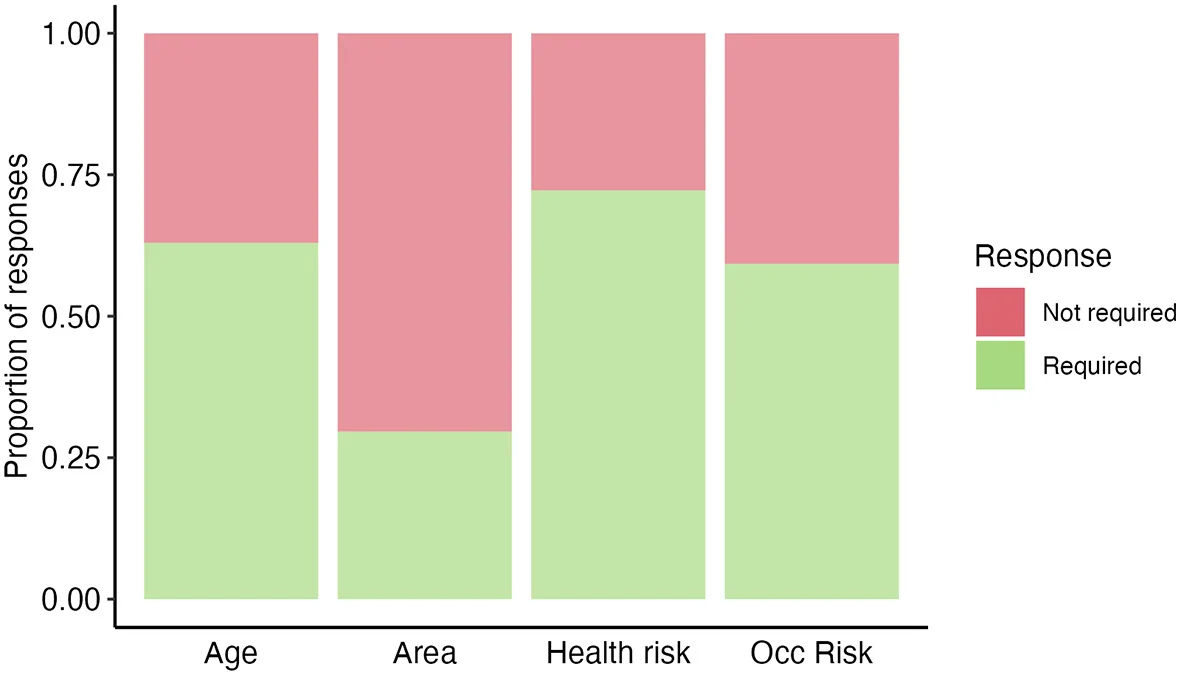
High Burden Country (HBC) experts' opinion (*n*=54) on “*If a new TB vaccine was introduced, how would your country prioritize potential population groups?*”. Answers were recorded based on a respondent's opinion on whether a category was useful/required for prioritizing (required—green) or not useful/not required for prioritizing (not required—red) potential population groups. Occ risk, occupational risk.

We report here the data from the surveys and breakout groups categorized by the data areas of epidemiology, impact, feasibility, and acceptability. The full country-level data are shown in the Table 1 and can be found online here. Data from the 54 respondents (both HBC and global experts) is included in the “Data from Survey Respondents” tab, with the “Data from Workshop Leads” tab containing knowledge on the availability of data for each country collated by workshop leads. Results are presented in Figures 1, 2, with Figures 1A, B, 2 generated based on 54 survey responses from HBC (data needs and availability) and global experts (data availability only). For Figure 1C, data from HBC and global experts were combined with the additional knowledge on data availability for each country from workshop leads. We added an additional category, “Additional insights emerging from the workshop,” which emerged as cross-cutting themes during the breakout group sessions.

**Table 1:** Full High Burden Country (HBC)-level data on data needs and availability for the 49 countries on at least one of the three WHO high TB burden lists (6), as well as Afghanistan, Cambodia, Taiwan, and The Gambia. (Table key below).

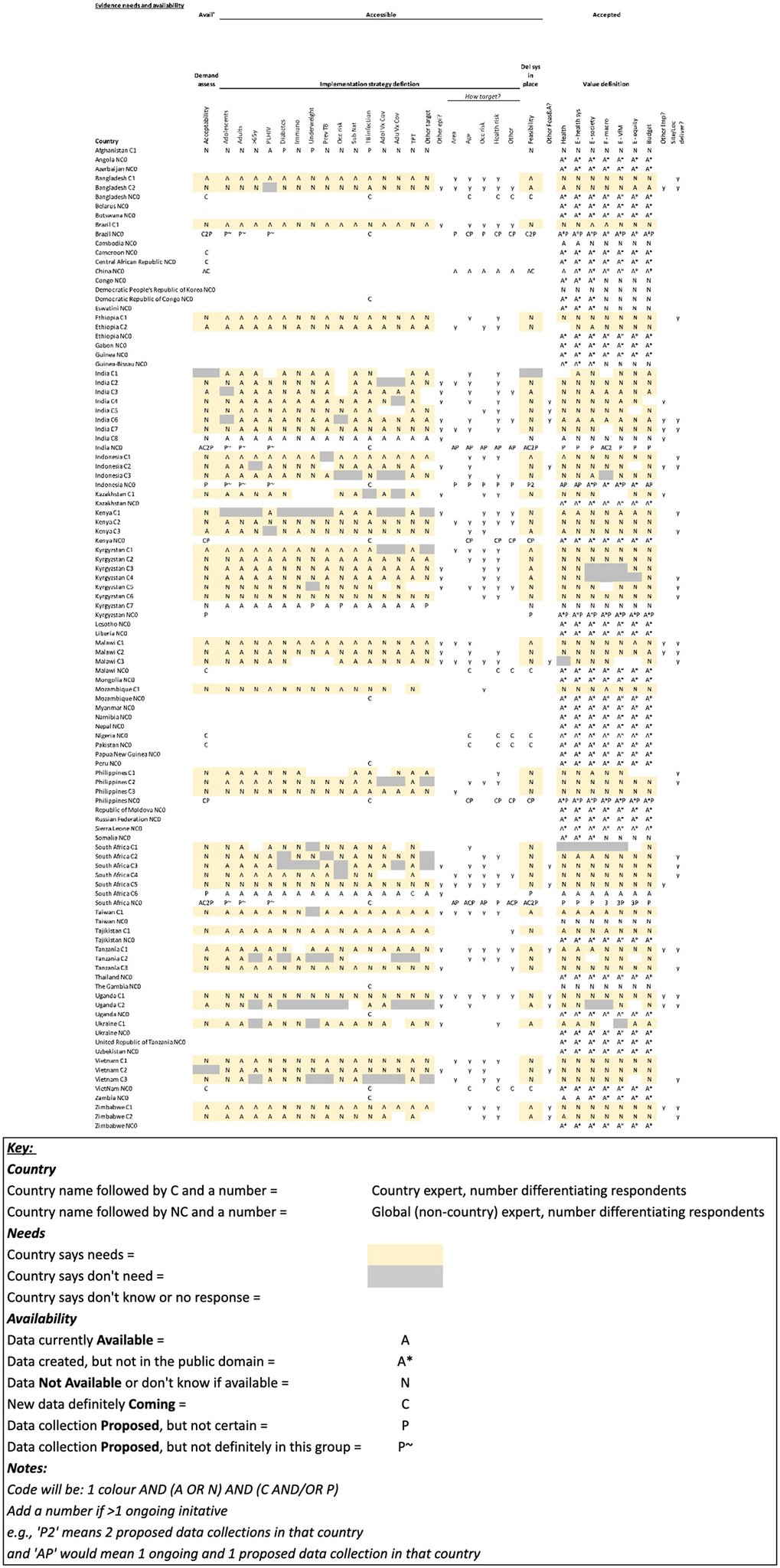

PLHIV, people living with HIV; Immuno, immunocompromized individuals; Prev TB, individuals with previous tuberculosis disease; Occ risk, occupational risk; Sub Nat, subnational level data; Adol Vx Cov, adolescent vaccine coverage; Adu Vx Cov, adult vaccine coverage; TPT, TB preventive therapy coverage.

### 3.2 Epidemiology

#### 3.2.1 Epidemiological data for decision making—Needs

In the pre-workshop survey, the majority of HBC experts (~60%) expressed a need for all the specified epidemiological data (Figure 1A). This included data on potential priority group sizes and TB burdens in specific groups, as well as data on TB infection prevalence, coverage of other vaccines and TPT (Figure 1A). The highest expressed epidemiological data need was for population size and TB burden data on adults and sub-nationally, for TB infection prevalence data and TPT coverage data. HBC experts expressed the lowest need for population size and TB burden data on over 65-year-olds, populations with underweight, those with previous TB, occupational risk groups, and vaccine coverage data.

#### 3.2.2 Epidemiological data needed for decision making—Availability and gaps

We then asked HBC and global experts if they already had the epidemiological data the HBC experts said they needed. In the pre-workshop survey, HBC and global experts expressed greatly varying availability of epidemiological data (Figure 1B). Over 50% of HBC and global experts reported the current availability of existing population size/TB burden data on adolescents, adults, over 65-year-olds, people living with HIV (PLHIV), and people with previous TB, sub-national data, TB infection prevalence, adolescent vaccine coverage, and TPT coverage. Low data availability was reported (<39% of HBC and global experts) on individuals with diabetes, immunosuppression, underweight, occupational risk, and adult vaccine coverage.

Data collated by workshop leads identified additional ongoing studies to collect additional TB infection prevalence data for 14 countries (South Africa, Mozambique, Zambia, Democratic Republic of the Congo, The Gambia, Kenya, Uganda, The Philippines, Indonesia, Vietnam, Bangladesh, India, Brazil, and Peru) by GMRI. Experts also reported future plans, in some cases contingent on funding, to collect TB burden data on adolescents, adults, PLHIV, and underweight individuals (Figure 1C). These may be collected by IAVI, the US National Institutes of Health, and KNCV.

HBC and global experts in the breakout groups reported that, in the context of resource constraints, it will be important to define priority target populations for the initial introduction of new TB vaccines. It was discussed that data collection should be disaggregated by key demographics to better describe local epidemics and inform target populations. Workshop participants proposed that epidemiological and household surveys should be prioritized to provide up-to-date data on the TB burden among target populations. These data should be incorporated into future economic and public health modeling studies to support decision-making.

### 3.3 Impact

#### 3.3.1 Health and economic Impact data for decision making—Needs

In the survey, more than 74% of HBC respondents expressed a need for all the specified health and economic impact data (Figure 1A). This included data on the impact of vaccines on health, health systems, society, macroeconomics, equity, and budgets, and the value for money of new vaccines. The highest expressed need was for evidence on the impact on health, health systems and budgets, and the lowest on macroeconomics and equity.

#### 3.3.2 Health and economic Impact data needed for decision making—Availability and gaps

HBC and global experts reported very low availability for all health and economic data (fewer than 24% of all respondents, Figure 1B). However, workshop leads were aware of some available health and economic impact data, including detailed country-specific modeling impact data for India, South Africa, China, Indonesia, Cambodia, and Zambia (7–15), and modeling results for 105 individual countries that were used to inform the WHO TB Vaccine Investment Case (Figure 1C) (16–19). The latter results have been released at a regional, income group, and TB burden level, but have not been released at the country-level. The country-level results can be accessed by countries in discussion with the authors (16–19).

HBC and global experts in the breakout groups said country-specific health impact and cost-benefit analyses should be conducted that capture the return of investment at health systems and micro- and macro-economic levels. Workshop participants emphasized that economic models should assess key domains influencing decision-making, including delivery costs and impact on equity-related domains, such as out-of-pocket expenses, catastrophic household costs, and social protection programs. Where possible, these exercises should assess the budget impact at the ministerial, NTP, and subnational levels. Parallel demand and market forecasting can inform decisions on financing for procurement and implementation and build a stronger case for return on investment for industry partners. Resource gaps should also be mapped to inform domestic and donor investment strategies.

The experts recommended that as new data become available, they should be used to update existing country-level health and economic impact modeling to better inform decision-making, and these models should be applied to a wider range of countries, in partnership with those countries, so this information is available when required by HBCs.

### 3.4 Feasibility

#### 3.4.1 Feasibility data for decision making—Needs

Feasibility was described as any data relating to the practicality of vaccine implementation, concerning logistical, delivery, and program-related considerations. In the survey, the majority of HBC experts (88%) expressed a need for feasibility data (Figure 1A).

#### 3.4.2 Feasibility data needed for decision making—Availability and gaps

HBC and global experts expressed very low availability of feasibility data, with <29% of respondents indicating the availability of these data (Figure 1B). Workshop leads were aware that some data on feasibility will be collected in five countries by SMART4TB, beginning in Kenya and South Africa (Figure 1C).

While the survey did not collect responses on disaggregated categories of feasibility data, the participants in the workshop breakout groups provided insight into key data gaps and barriers to successful vaccine implementation faced at the country level. Participants expressed that, at present, there is little alignment between country-level stakeholders on delivery scenarios and strategies to reach target populations. Participants also reported multiple constraints in manufacturing, procurement, delivery, and regulatory pathways and infrastructure. Barriers included limited regional manufacturing capacity, low affordability for self-procurement of vaccines by non-Gavi eligible countries, and lack of infrastructure to maintain the vaccine cold chain. Intellectual property barriers, including patents and terms of licensure that extend periods of market exclusivity, were a noted concern for access. A lack of clear timelines on when vaccines will be available, and the resources needed to support their implementation emerged as additional barriers to preparedness activities. The lack of certainty on the number of doses and timing of dose administration of a future TB vaccine was also highlighted as a barrier.

HBC and global experts in the breakout groups suggested that potential implementation strategies should be integrated into national strategic plans, immunization programs, and applicable health services ahead of market entry, and guidance should be developed to support this with consensus between stakeholders involved. Participants noted that these plans should identify how to reach target populations, acknowledging that the strategies will vary by country, depending on factors including local epidemiology, immunization and TB control programs, and infrastructure. Participants highlighted that lessons from the introduction of other vaccines for adolescents and adults, such as HPV and COVID-19 vaccination, should also be applied. Further, participants advised that where required, feasibility studies should be incorporated into country-level strategic plans and that they should be costed appropriately.

### 3.5 Acceptability

#### 3.5.1 Acceptability data for decision making—Needs

In the pre-workshop survey, the majority of HBC experts (88%) expressed a need for acceptability data (Figure 1A), described as the acceptability of the vaccine to the potential target populations and other key stakeholders.

#### 3.5.2 Acceptability data needed for decision making—Availability and gaps

HBC and global experts expressed very low availability of acceptability data, with only 17% of respondents indicating availability (Figure 1B). Workshop leads were aware that some data on acceptability will be collected in five countries by SMART4TB, beginning in Kenya and South Africa (Figure 1C).

HBC and global experts in the break-out groups said that post-COVID, there is an increasing need for data on acceptance, including barriers to acceptability, such as vaccine hesitancy, mistrust, and misinformation among different populations. Further, participants reported that there is limited information on how to generate demand for new vaccines among adolescents and adults before market introduction, alongside varying health literacy levels among stakeholder groups and the challenges of reaching certain populations, such as people living in rural areas and migrant populations. Limited data were also reported to be available on vaccine hesitancy. It was also noted that prospective acceptability work can be challenging as the specific characteristics of products that will be licensed are not yet known.

HBC and global experts in the breakout groups said acceptability assessments should be carried out, be people- and community-centered, and have a broad representation of stakeholders, including religious and traditional leaders. Participants suggested that lessons should also be applied from existing vaccine efforts and health initiatives, while product-specific surveys may be useful once vaccine characteristics are better known. Participants emphasized that public health education and awareness building need to be tailored to the different stakeholder groups engaged and that vaccine benefits need to be clearly articulated and barriers to uptake need to be addressed, such as concerns about side effects and misunderstandings of vaccine efficacy in preventing disease vs. infection.

Further, breakout group participants felt diverse approaches should be used to address vaccine acceptability issues, including in-person outreach campaigns, social media, and print media. In support of this, participants recommended that media specialists, including data journalists, should be trained in reporting on TB vaccines and that researchers should be trained to communicate across different platforms, while respected public health experts, politicians, and policymakers can be sensitized and leveraged as champions to build awareness.

### 3.6 Additional insights emerging from the workshop

Analysis of the HBC and global experts' feedback identified needs that cut across the four domains of epidemiology, impact, feasibility, and acceptability.

There were inconsistencies in the knowledge of existing data expressed by HBC experts. In the pre-workshop survey, the same type of data (i.e., within the same column) was reported as being both available (A) and not available (N) by different HBC experts in the same country, e.g., see data on India and South Africa in the Table 1. These data awareness differences between HBC experts can be seen in multiple countries (Table 1). This finding was supported by discussions in the workshop.

There was also a lack of knowledge by HBC and global experts of existing data created in globally led activities. For example, of the health and economic impact data created for the WHO TB Vaccine Investment Case (16–19).

HBC and global experts were also largely unaware of ongoing and newly proposed data collection activities. Indian and South African participants reported there were at least two ongoing initiatives to collect new acceptability and feasibility data, and at least one new initiative proposed to collect new data. In South Africa, Kenya, Zambia, the Philippines, the Kyrgyz Republic, and Pakistan, proposed/planned data collection efforts were also identified. Other workshop attendees were largely unaware of these activities.

In the breakout groups, some reasons for this lack of awareness were proposed. HBC and global expert participants noted that data collection is often resource-constrained, with limited numbers of trained personnel, limiting data sharing, and that there was sometimes a lack of transparency in the private sector. Experts noted that these data awareness differences may lead to duplicative and unstandardized data collection within and between countries, resulting in suboptimal use of limited resources. It is very possible that valuable data sources remain unknown and unexploited, and this could help address existing data gaps. HBC and global expert participants also commented that the data available were often not sufficiently relevant or recent.

The participants recommended that the availability of existing and prospective data should be mapped and shared, and that standardized data collection and data management systems should be established at the sub-national, national and global levels in preparation for TB vaccine introduction. Further, the participants noted that transparent data collection and analysis, and dissemination of evidence by all relevant stakeholders, will be required to effectively inform TB vaccine introduction strategies and decision-making.

### 3.7 TB vaccine priority groups

In the survey, we asked HBC experts to identify what target populations they would consider prioritizing if they were to introduce TB vaccines to adolescents/adults (Figure 2).

HBC respondents proposed that they would likely prioritize by age (e.g., the elderly), occupational risk group (e.g., miners and healthcare workers), and health risk groups (e.g., immunocompromized and malnourished individuals). Less commonly, respondents said they would prioritize by geographic area. Other suggested target populations reported in the survey included prison populations, household contacts, and underrepresented populations.

## 4 Discussion

HBC experts expressed a high demand for epidemiological, impact, feasibility, and acceptability data. They reported variable availability of existing epidemiological data, and low availability for impact, feasibility, and acceptability data. Workshop leads reported additional knowledge of existing data in some HBCs on impact, upcoming data collection on infection prevalence, acceptability and feasibility data, and the potential of new epidemiological data collection on adolescents, adults, people living with HIV and underweight individuals.

HBC and global experts made key recommendations on creating a robust, harmonized and coordinated data collation, collection, analysis and sharing system at subnational, national and global levels; using new data to update existing country estimates of health and economic impact and extend analyses to a wider range of countries; carrying out demand and market forecasting; resource gap mapping; work to align delivery strategies; work to address manufacturing, procurement, delivery, and regulatory barriers; sharing the timing of potential vaccine licensure; incorporating TB vaccine introduction strategies into national strategic plans, immunization programs, and applicable health services; collecting data on vaccine hesitancy, mistrust, and misinformation; collecting data on how to generate adolescent/adult vaccine demand, and making increased funding available for these activities.

The experts also recommended expanding this analysis to other areas of the WHO framework, including more HBC stakeholders, and repeating this analysis after country and community advocacy/socialization around different candidates.

Our work complements ongoing endeavors by SMART4TB, IAVI, LSHTM, WHO, and other stakeholders, provides relevant information for funding allocation decision-making, and supports the need for additional coordination. By encouraging country ownership, this work can help stimulate demand at both global and HBC levels. Additionally, our work serves as a foundational reference that provides countries with a preliminary map of evidence needs and gaps to support the introduction of future new vaccines and facilitating strategic spending decisions.

We recognize that this effort has several limitations. The workshop evaluation involved only a subset of countries, limiting its generalizability. While we strived to be purposive in the selection of individuals invited to take part, participation followed a convenience sampling approach based on the availability of participants. As such, the HBCs and global organizations included were not fully representative. In addition, the survey tool deployed was not validated, and its reliability has not been verified. Moreover, the study was not designed to assess relative prioritization across different areas of evidence or types of data. Further work is needed to tease out which evidence gaps would be most pressing to address in specific contexts. The two breakout group sessions held during the workshop had broad and varied stakeholder representation between groups, which may limit the generalizability and saturation of the data. In light of these limitations, the interpretation of the workshop outcomes should be taken with caution. Forthcoming work should build upon and validate these preliminary findings.

We conclude that robust, harmonized and coordinated data collation, collection, analysis, and sharing systems are needed and should be adequately resourced to ensure that HBC and global stakeholders can use limited resources most efficiently, and that HBCs will have the data they need to decide whether to introduce new TB vaccines and to guide strategies for their efficient introduction. This approach should be repeated for other areas of the WHO framework, alongside expanding this analysis to include more stakeholders in each of the HBCs and repeating this analysis in the future after country and community advocacy/socialization around different candidates.

## Data availability statement

Original datasets are available in a publicly accessible repository: The original contributions presented in the study are publicly available. This data can be found here: https://docs.google.com/spreadsheets/d/1PIYMSz38F5vFlbgp1NDWyrDuqeST0e3GB4Nl71kCf3w/edit#gid=103346952 and in the article/Supplementary material.

## Ethics statement

Ethical approval was not required for the studies involving humans because all participants and respondents were informed before the meeting that anonymized data collected during the meeting or from the survey could be used in future reports and/or publications, and that participating in the meeting assumed consent for this data to be used. The studies were conducted in accordance with the local legislation and institutional requirements. The participants provided their written informed consent to participate in this study. Written informed consent was obtained from the individual(s) for the publication of any potentially identifiable images or data included in this article.

## Author contributions

RC: Writing – original draft, Writing – review & editing. CY: Writing – original draft, Writing – review & editing. SP: Writing – original draft, Writing – review & editing. EA: Writing – original draft, Writing – review & editing. SM: Writing – review & editing. RL: Writing – review & editing. BG: Writing – review & editing. LS: Writing – review & editing. GV: Writing – review & editing. ET: Writing – review & editing. MF: Writing – review & editing. IE: Writing – review & editing. AD: Writing – review & editing. TN: Writing – review & editing. TVCDNG: Writing – review & editing. PP: Writing – review & editing, Writing – original draft. RW: Writing – original draft, Writing – review & editing.

## Group member of TB Vaccine Country Data Needs Group

Pei-Chun Chan, The Division of Chronic Infectious Disease, Centers for Disease Control, Taiwan; Salome Charalambous, Aurum Institute, South Africa; Gavin Churchyard, Aurum Institute, South Africa; Alberto Garcia-Basteiro, Centro de Investigação em Saúde de Manhiça, Mozambique; Mustapha Gidado, KNCV Tuberculosis Foundation, The Netherlands; Michelle M. Gill, Elizabeth Glaser Pediatric AIDS Foundation, United States of America; Willem Hanekom, Africa Health Research Institute, South Africa; Mark Hatherill, University of Cape Town, South Africa; Benjamin Kagina, University of Cape Town, South Africa; Gulmira Kalmambetova, National TB Programme, The Kyrgyz Republic; Andrew D. Kerkhoff, Division of HIV, Infectious Diseases and Global Medicine Zuckerberg, San Francisco General Hospital and Trauma Center, University of California San Francisco, United States of America; Shodmon Khushvakhtov, KNCB TB PLUS, The Republic of Tajikistan; Mmamapudi Kubjane, Health Economics and Epidemiology Research Office (HE2RO), Wits Health Consortium, University of the Witwatersrand, Johannesburg, South Africa; Sandip Mandal, John Snow India, India; Leonardo Martinez, Department of Epidemiology, School of Public Health, Boston University, Boston, Massachusetts, United States of America; Harriet Mayanja-Kizza, Makerere University, Uganda; Dingaan Mithi, Journalists Association Against AIDS, Malawi; Abu Syed Md. Mosaddek, Department of Pharmacology, Uttara Adhunik Medical College, Uttara, Dhaka & WHO Snakebite envenoming Roster of Experts & Quest Bangladesh Lalmatia, Dhaka, Bangladesh; Lillian Mtei, KNCV TB PLUS, Tanzania; Bakyt Myrzaliev, KNCV TB PLUS, The Kyrgyz Republic; YaDiul Mukadi, United States Agency for International Development, United States of America; Tom Rogers Muyunga-Mukasa, Advocacy Network Africa, Kenya; Patricia Asero Ochieng, CEO, Ringa Women Fighting AIDS Group, Kenya; Surabhi Pandey, Public Health Foundation of India, India; Awnish Singh, Department of Epidemiology, School of Public Health, University of Michigan, United States of America; Former, National Technical Advisory Group on Immunization Secretariat, Ministry of Health and Family Welfare, India; Divya Kantilal Shah, Wellcome, United Kingdom; Stephen Anguva Shikoli, Pamoja TB Group, Kenya; Edina Sinanovic, University of Cape Town, South Africa; Michele Tameris, South African Tuberculosis Vaccine Initiative, University of Cape Town, South Africa; Kitaw Teklemariam, Organic Health Care Service, Ethiopia; Edna Tembo, Coalition of Women & Girls Living with HIV & AIDS, Malawi; Jaspreet Turner, Wellcome, United Kingdom; Jenny A. Walldorf, World Health Organization, Switzerland; Jennifer Woolley, Stop TB Partnership Working Group on New TB Vaccines, New York, NY, United States.
