# Supplementary material for: Bridging the gap: evaluating high TB burden country data needs to support the potential introduction of TB vaccines for adolescents and adults: a workshop report
Source: Front Tuberc. 2024 Apr 29;2:1384036. doi: 10.3389/ftubr.2024.1384036 (PMC13494980; doi:10.3389/ftubr.2024.1384036)
Supplement: Supplementary file 1 [file Data_Sheet_1.PDF]

Stop TB Partnership

Working Group on New TB Vaccines

WGNV Pre-Workshop Survey

As a representative of a country that may consider rolling out new TB vaccines to adolescents and/or adults, we are asking you to say which key pieces of TB vaccine epidemiological, health and economic impact, feasibility and acceptability data, your country would need to support its decision-making. IE to consider:

If, and how, does your country roll out new or repurposed TB vaccines to an adolescent and/or adult population?

Note, this survey is designed to just cover country level epidemiological, health/economic impact, feasibility and acceptability data, so for example, you can assume you will also have licensure trial data on vaccine efficacy and safety.

Data will be anonymized, and summary data will be available to meeting participants, implementing partners, and online.

For questions please reach out to: richard.white@lshtm.ac.uk

Sign in to Google to save your progress. Learn more

* Indicates required question

Please enter your name: *

This will not be shared publicly, and will only be used to follow up, eg for clarification.

Your answer

Please enter your email address: *

This will not be shared publicly, and will only be used to follow up, eg for clarification.

Your answer

What country are you reporting on? (if multiple please fill out another form) *

Choose

If this country was going to introduce TB vaccines to adolescents/adults, do you think they would consider targeting based on: ?

- [ ] Geographic area
- [ ] Age
- [ ] Occupational risk (eg. miners, health care workers)
- [ ] Health risk (eg immunocompromised, malnourished)
- [ ] Other: ____________________

In your expert opinion, are these data below needed in this country's decision making? And if so, are these data available?

Please complete the 3 tables below, selecting only one option per row.

Epidemiological Data *

	NOT NEEDED for decision-making	NEEDED for decision-making AND WE HAVE these data	NEEDED for decision-making BUT I DON'T KNOW IF WE HAVE these data	I don't know
Population size and TB burden of adolescents (12 - 18 years)	○	○	○	○
Population size and TB burden of adults (18-64 years)	○	○	○	○
Population size and TB burden of persons older than 65 years	○	○	○	○
Population size and TB burden of persons living with HIV	○	○	○	○
Population size and TB burden of persons living with diabetes	○	○	○	○
Population size and TB burden of immunocompromised persons	○	○	○	○
Population size and TB burden of underweight persons	○	○	○	○
Population size of population of people who have previously had TB disease	○	○	○	○

Population size and TB burden of occupational risk groups (eg. miners, health care workers)	○	○	○	○
Population size and TB burden, at subnational level	○	○	○	○
Population size and TB burden of other potential target populations (please specify below)	○	○	○	○
TB infection prevalence	○	○	○	○
Coverage of other adolescent vaccines (if any other vaccines given in country)	○	○	○	○
Coverage of other adult vaccines (if any other vaccines given in country)	○	○	○	○
TPT coverage	○	○	○	○

Are there other epidemiological data that you think are needed or have that will help inform the decision-making?

Would you like to make any other comments about epidemiological data?

Your answer

Health and economic impact data *

	NOT NEEDED for decision-making	NEEDED for decision-making AND WE HAVE these data	NEEDED for decision-making BUT I DON'T KNOW IF WE HAVE these data	I don't know
Health impact (eg number of cases and deaths averted by vaccine)	○	○	○	○
Economic impact - on health systems (ie costs incurred or saved by TB and/or immunization programmes)	○	○	○	○
Economic impact - on society (ie costs on patients due to disease or accessing care, and/or productivity losses, and/or catastrophic health costs on households)	○	○	○	○
Economic impact - macroeconomic (eg on country economic growth or employment)	○	○	○	○

Value for money (eg cost per death or DALY averted)	○	○	○	○
Budget impact (total cost of introduction and delivery of programme)	○	○	○	○
Impact on equity	○	○	○	○

Are there other health and economic impact data that you need or have that will help inform your decision-making?

Would you like to make any other comments about health and economic impact data?

Your answer

Feasibility and acceptability data *

	NOT NEEDED for decision-making	NEEDED for decision-making AND WE HAVE these data	NEEDED for decision-making BUT I DON'T KNOW IF WE HAVE these data	I don't know
Feasibility (ie practicality of vaccine implementation, considering the logistics, delivery, and program related considerations)	○	○	○	○
Acceptability (ie the acceptability of the vaccine to the target populations and other key stakeholders)	○	○	○	○

Are there other feasibility and acceptability data that you need or have that will help inform your decision-making?

Would you like to make any other comments about feasibility and acceptability data?

Your answer

Do you have any thoughts on the preferred site/location of delivering vaccines for key target groups?

Your answer

This is the end of the survey.

Thank you for taking part.

Do you have any other comments?

Your answer
